# Dogs can be trained to find a bar magnet

**DOI:** 10.7717/peerj.6117

**Published:** 2018-12-17

**Authors:** Sabine Martini, Sabine Begall, Tanja Findeklee, Marcus Schmitt, E. Pascal Malkemper, Hynek Burda

**Affiliations:** 1 Department of General Zoology, Faculty of Biology, University of Duisburg-Essen, Essen, Germany; 2 Department of Game Management and Wildlife Biology, Faculty of Forestry and Wood Sciences, Czech University of Life Sciences, Prague, Czech Republic; 3 Dog School Findeklee, Essen, Germany

**Keywords:** Magnetoreception, Domestic dogs, Behavioral test, Operant conditioning

## Abstract

Magnetoreception, the ability to sense the Earth’s magnetic field (MF), is a widespread phenomenon in the animal kingdom. In 1966, the first report on a magnetosensitive vertebrate, the European robin (*Erithacus rubecula*), was published. After that, numerous further species of different taxa have been identified to be magnetosensitive as well. Recently, it has been demonstrated that domestic dogs (*Canis lupus familiaris*) prefer to align their body axis along the North–South axis during territorial marking under calm MF conditions and that they abandon this preference when the Earth’s MF is unstable. In a further study conducting a directional two-choice-test, dogs showed a spontaneous preference for the northern direction. Being designated as putatively magnetosensitive and being also known as trainable for diverse choice and search tests, dogs seem to be suitable model animals for a direct test of magnetoreception: learning to find a magnet. Using operant conditioning dogs were trained to identify the MF of a bar magnet in a three-alternative forced-choice experiment. We excluded visual cues and used control trials with food treats to test for the role of olfaction in finding the magnet. While 13 out of 16 dogs detected the magnet significantly above chance level (53–73% success rate), none of the dogs managed to do so in finding the food treat (23–40% success rate). In a replication of the experiment under strictly blinded conditions five out of six dogs detected the magnet above chance level (53–63% success rate). These experiments support the existence of a magnetic sense in domestic dogs. Whether the sense enables dogs to perceive MFs as weak as the Earth’s MF, if they use it for orientation, and by which mechanism the fields are perceived remain open questions.

## Introduction

Magnetoreception has been reported for a variety of animal taxa from arthropods to vertebrates (Wiltschko & Wiltschko, 1995). However, most studies have focused on homing pigeons, migratory birds, and migratory turtles. Magnetoreception in other species and other behavioral contexts than long-distance navigation and homing has remained understudied. Correspondingly, most evidence for magnetoreception is based on behavioral studies (observations and experiments) using orientation assays such as recording preferred directions of stereotype movement (“Zugunruhe”) in Emlen funnels (Emlen & Emlen, 1966; Mouritsen et al., 2009; Muheim, Moore & Phillips, 2006b; Ritz et al., 2004; Wiltschko & Wiltschko, 2003). In rodents, the so-called nest-building assay has been successfully applied (Burda et al., 1990; Malkemper et al., 2015; Marhold, Wiltschko & Burda, 1997; Muheim et al., 2006a; Oliveriusová et al., 2014; Painter et al., 2018) and several studies revealed the heuristic potential of magnetic alignment, a tendency to align the body axis with respect to magnetic field (MF) lines (Begall et al., 2008, 2013; Červený et al., 2011). All these assays make use of spontaneous directional reactions of animals.

In general, contrary to research on other sensory modalities, only a few studies of magnetoreception have made use of associative learning, that is, operant conditioning, and managed to train the investigated species to react with respect to magnetic anomalies or to learn directional information provided by MFs (Denzau et al., 2011; Dommer et al., 2008; Freire et al., 2012; Mora et al., 2004; Muheim et al., 2006a; Newton & Kajiura, 2017; Phillips, 1977; Vácha, Půžová & Drštková, 2008; Voss, Keary & Bischof, 2007; Walker & Bitterman, 1989; Wilzeck et al., 2010). The failure to condition animals on magnets, which constitute magnetic anomalies, is related to the problem that animals, which use magnetoreception for long-distance orientation, might have difficulties in associating magnetic cues with, for example, a reward in a small scale of a laboratory cage (cf. Wiltschko & Wiltschko, 1996). Most scholars (and nonspecialists) will presumably consider the ability to associate a magnetic cue with a reward to be the ultimate evidence of magnetoreception, much like psychophysics is the gold standard to study the relationship between a physical stimulus and its sensory perception.

Recently, it has been suggested that domestic dogs perceive the Earth’s MF and are responsive to its small variations (Hart et al., 2013b). Under calm MF conditions, the dogs showed a consistent magnetic alignment and preferred to mark their home ranges with the body being aligned roughly along the North–South axis. During unstable MF conditions and particularly during geomagnetic storms this directional preference was abolished. We assume that compass alignment (requiring *physical* body rotation) facilitates the organization and reading of a mental map (which otherwise would require *mental* rotation). In a subsequent study, we could demonstrate that domestic dogs have a spontaneous directional preference. Here, the animals had to choose between two dishes with snacks that were placed left and right in front of them, in two neighboring compass directions (N vs E, E vs S, S vs W, and W vs N). Although the laterality of respective dogs primarily determined their choice, there was a preference for the dish placed north (“pull of the north”), which was particularly strong in the test combination north–east (Adámková et al., 2017). Even though the biological meaning of magnetic alignment remains speculative and the sensory mechanism behind it remains unknown, the (probable) existence of magnetoreception in dogs is intriguing and, if true, would stimulate further research and would also open up a variety of new research possibilities. Dogs are well known for their cognitive abilities and capability of being trained to react on a variety of signals and cues (visual, acoustic, olfactory, tactile) (Adachi, Kuwahata & Fujita, 2007; Cornu et al., 2011; Ehmann et al., 2012; Miklósi, 2015; Müller et al., 2015) and thus would represent a promising animal model to unravel the intricate details of the (mammalian) magnetic sense. Therefore, we tested whether dogs could be trained to detect MFs.

## Materials and methods

Dogs were trained in two test series to identify and point at a bar magnet (test series A: treatment) or a food treat (test series B: control) using operant conditioning with positive reinforcement. Both test series consisted of four training phases of increasing difficulty and the three-alternative forced-choice experiment, with the magnet or the food treat hidden in one of three jars. Test series B was performed to determine the potential role of olfaction in the general experimental setup. An additional experiment (replicate of test series A) has been performed in a truly blind fashion to test a possible Clever Hans effect.

Altogether 18 dogs (11 males, seven females), aged 2–13 years, of different breeds, passed the training and completed the testing (Table 1). Two dogs participated only in test series A, two other dogs only in test series B, and the 14 remaining dogs in both test series. All of these 14 dogs, except one (Hy), were trained and tested in test series A (magnet) before test series B (food treat). Finally, six of the dogs were also tested in the blind replication experiment. The whole study was conducted in the open field between spring 2015 and winter 2017 with altogether 1,110 executed and analyzed trials. Different dog owners and five experimenters performed both test series.

**Table 1:** Overview of the tested dogs and their data.

Dog	Breed	Sex	Age	Size category	*n* magnet (test series A)	Correct choices magnet (blind replicate) (%)	*n* food treat (test series B)	Correct choices food treat (open jars) (%)
Dt	Border collie cross	M	6	L	30	57 (53)	30	33
Gy	Jack russel	M	6	M	30	53	30	37
Hy	Chihuahua	M	5	S	30	37	30	23 (90)
Jk	Jack russel	M	13	M	30	67	–	
Jy	Retriever cross	M	3	L	30	43 (57)	30	40
Ka	Crossbreed	M	10	L	30	63 (60)	30	33
Kr	Australian shepherd	M	8	L	30	53	30	30
Mx	Retriever	M	5	L	30	53	30	37
Pl	Retriever	M	2	L	30	63	30	30 (77)
Sy	Retriever cross	M	3	L	30	53 (63)	30	30
Ts	Terrier cross	M	3	S	30	60	30	30
Bi	Dachshund cross	F	3	S	30	67 (60)	30	37 (83)
Ca	Grand basset griffon vendeen	F	10	M	30	73	30	30
Fi	Terrier cross	F	3	S	30	57	30	40
Le	Retriever	F	7	L	30	47		
Ly	Jack russel—dachshund cross	F	4	M	30	73	30	40
Pa	Collie cross	F	9	L			30	23 (87)
Ya	Gos d’aturo catala cross	F	2	L			30	33

**Notes:**

Dogs’ names are abbreviated by two letters; dogs are sorted by sex and arranged alphabetically by their abbreviated names.

Sex: F, female, M, male; age is given in years; size with regard to withers height: s, small, m, medium, l, large (cf. FCI classifications); *n*, number of trials; percentages for correct choices are rounded (with chance level at 33.3%).

To condition the dogs, we used a commercial clicker. We used three identical brown, fully opaque, wide-necked glass jars (dimensions: volume 100 ml, diameter 49 mm, height 96 mm, closed with tightly sealing polypropylene screw caps and additional polyethylene seals to protect the interior from air and moisture) for both test series.

In test series A, one of the three jars contained a ferrite bar magnet of 0.4 T remanence and the other two jars contained similar nonmagnetic brass objects. The jar containing the magnet and its position in the test row were randomized between the trials. The field strength of the magnet was approximately one microtesla (µT) at a distance of 40 cm, measured by a digital magnetometer (DTM-141, Group 3). At more considerable distances (> 40 cm) the magnet was detectable in the nanotesla (nT) range.

In test series B, a food treat preferred by the particular dog was hidden in one jar while the other two jars again contained nonmagnetic objects of similar shape and size.

All the items inside the jars were wrapped in black cotton socks. We adapted Gellermann’s rules, for example, no more than three times left, middle, or right in succession and well-balanced placement combinations, to guarantee random/prevent predictable orders of the alternating stimulus when changing the magnet’s or food treat’s position (Gellermann, 1933). Furthermore, we regularly exchanged the contents of the jars (on average after every third trial). Both the orientation of the jars (and thus the magnet) and the dog’s magnetic direction of movement (with respect to the geomagnetic field) toward the jars were randomized between all trials.

Both test series consisted of four training phases of increasing difficulty and the final experiment with a maximum of 30 trials each. In the first training phase, we established clicker training to make the dogs associate the magnet/food treat with a food reward. Subsequently, the dogs had to point at the jar containing the searched item—first in a single-choice-experiment (second training phase) and later in a two-choice-experiment (third training phase). In this phase of the experiment, three dogs were excluded from further procedure since they did not manage to decide on any of the jars without the help of a pointing gesture. Eventually, in the fourth training phase and the final experiment, the 18 remaining dogs should choose the magnet/food treat in a three-choice-experiment. The dogs’ choice included retrieving the jar, tapping at the jar or knocking over the jar using a paw or the snout (depending on the dog’s preference). In the single-choice-experiment (second training phase) the dogs were on average trained in 11 trials (at least at 2 days with a minimum of five trials per day). In the two- and three-choice-experiment (third and fourth training phase) each dog completed at least 25 trials (on not less than 5 days with a minimum of five trials per day) in both test series (magnet and food treat) before being tested.

**Figure 1:**
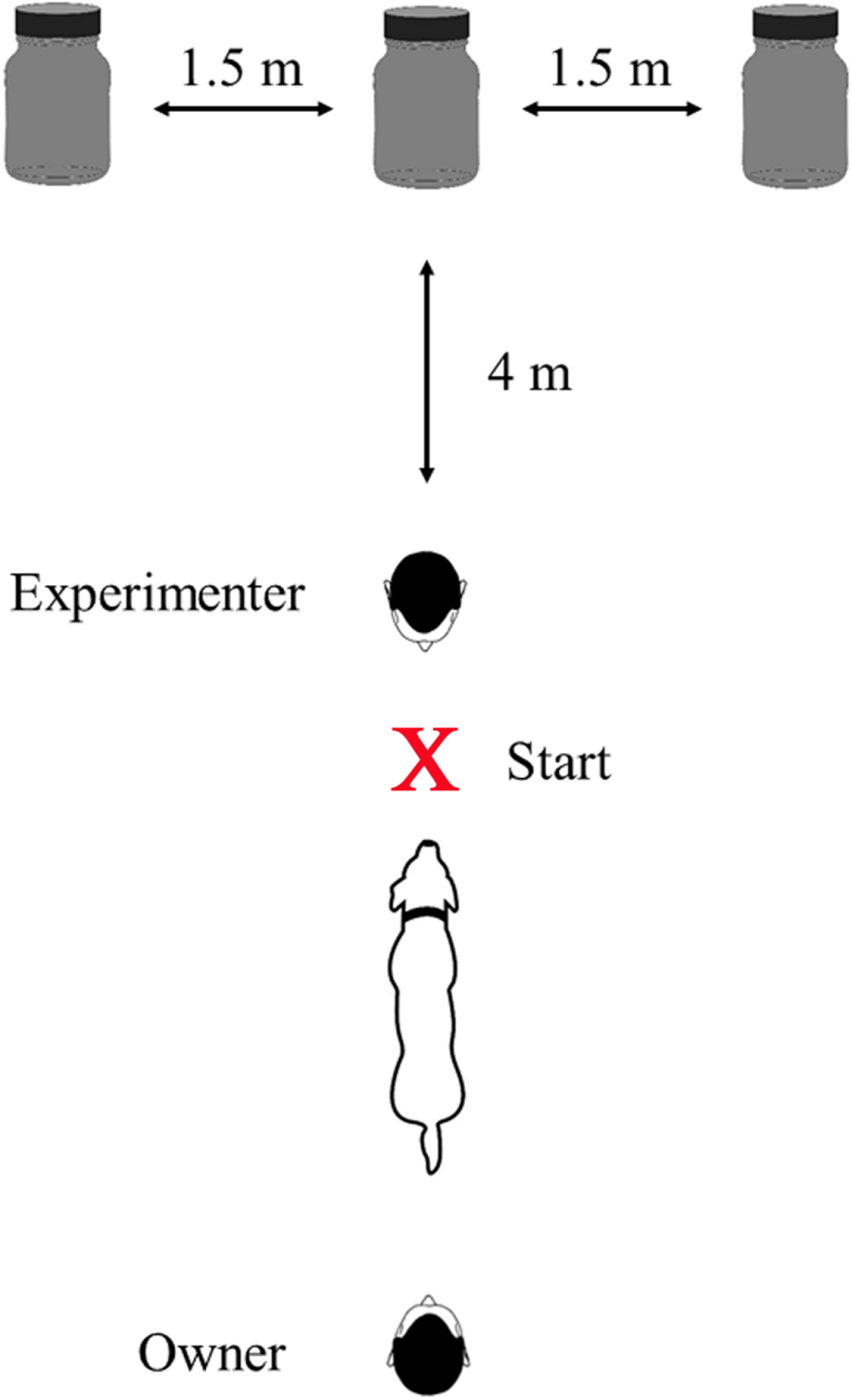
Top view of the experimental setup. Dimensions of the jars: volume 100 ml, diameter 49 mm, height 96 mm. In each trial, one of the opaque jars either contained a bar magnet or a dog treat. The X marks the starting point at which the search command was given.

The final experiment consisted of *n* = 30 trials each for the magnet series and the food treat series. In each trial, the experimenter lined up the jars at a distance of 4 m from the starting point to the middle jar (distance between the jars was 1.5 m). Both the owner and the dog could not observe the preparation procedure. Then the owner brought the dog to the starting point. Throughout the testing, the dog owner was standing behind the dog without having eye contact with the animal whereas the experimenter positioned herself/himself with the back to the jars directly in front of the dog (Fig. 1). The experimenter then gave the search command “Find the magnet” (test series A) or “Find the food treat” (test series B). After the dog had passed the experimenter, he/she turned around to observe the dog.

The blind replication experiment included a second experimenter and two custom-made partitions with three panels each (dimensions: width 3 × 55 cm, height 170 cm) which functioned as sight protection. Here, experimenter 1 prepared the setup while experimenter 2, the owner and the dog were waiting behind the second partition with no chance of seeing experimenter 1 or the setup (see Supplemental Information 1). After setup completion, experimenter 1 moved behind the first partition and experimenter 2 (who was blind to the contents of the jars) started the experiment as described above (see Supplemental Information 2). As soon as experimenter 2 got an acoustic signal from experimenter 1, who observed the dog’s performance through a small gap from behind the partition, he/she rewarded the dog immediately. Experimenter 1 did not have eye contact with anybody at any time of the experiment and touched all three jars equally while preparing the setup to prevent any olfactory clues.

Furthermore, four dogs were tested in an additional experiment where we removed the caps from the jars to test whether they are able to find the food treat when olfactory cues are available and whether they behave differently in finding the object.

In each trial, we recorded the first choice, and the experimenter immediately rewarded correct choices (magnet or food treat) with the clicker and a dog treat. In order to prevent the dogs from becoming frustrated or refusing the cooperation, they were allowed to make a second and third choice to get their reward, when necessary. Apart from the dog’s choices, we recorded the animals’ and owners’ identities, locality, place, date, time, and weather.

The difference between expected (chance level) and observed numbers of correct first choices were analyzed independently for each dog in each test series (magnet and food treat) by a Chi-square test. To test for significant differences between the number of correct choices in the two-test series (treatment and control) we used a paired sample *t*-test, to examine possible differences between sexes we used an unpaired *t*-test, and to check for age- and size-dependent differences we performed ANOVAs. We compared the mean success rate of all dogs in each test series with the expected success rate at chance level (33.3%) with a one sample *t*-test. The number of correct choices in each test series were normally distributed (Shapiro–Wilk test, *p* > 0.05). We conducted all analyses with SPSS 24.0.

## Results

### Test series A

**Figure 2:**
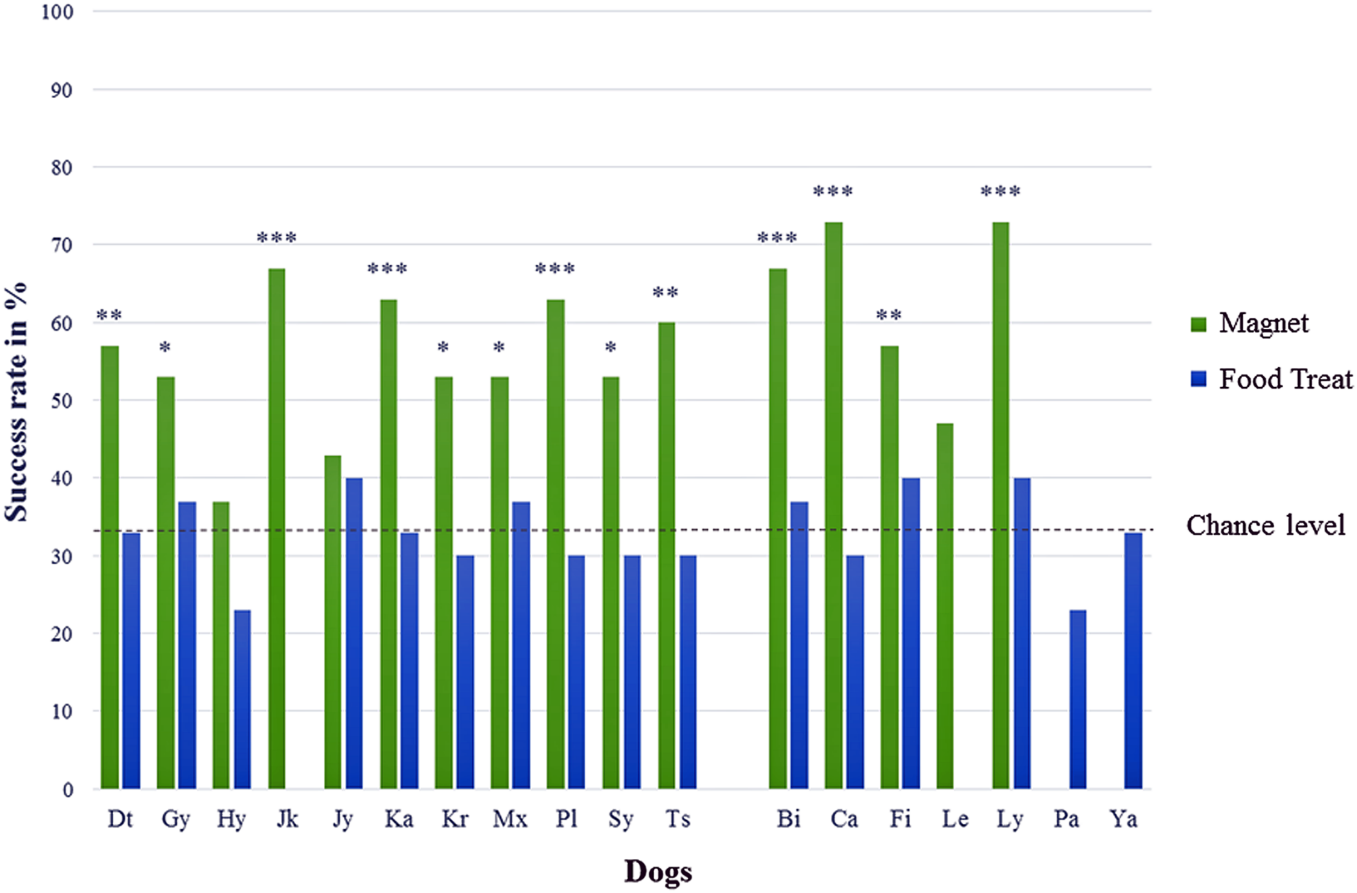
Percentage of correct choices for the tested dogs. Names are abbreviated by two letters; males are on the left, females on the right; both sexes are sorted by their abbreviated names in test series A (magnet) and test series B (food treat); with *p < 0.05, **p < 0.01, and ***p < 0.001 (chance level at 33.3 %).

In test series A (“Find the magnet”), 13 out of 16 dogs showed a significantly correct choice (Chi-square test performed individually for each dog with all 13 *p*-values ≤ 0.02) for the jar with the bar magnet and the success rates of those dogs were on average 61 ± 7% ranging between 53% and 73% (cf. Table 1, chance level at 33.3% and Fig. 2). The mean success rate of all 16 dogs differed significantly from the expected success rate at chance level (one sample *t*-test, *T* = 9.472, *p* < 0.001). No significant differences could be identified within the test group with respect to sex (unpaired *t*-test, *T* = 1.621, *p* = 0.353), age (ANOVA, *F* = 1.429, *p* = 0.275), and body size (ANOVA, *F* = 2.619, *p* = 0.111).

**Figure 3:**
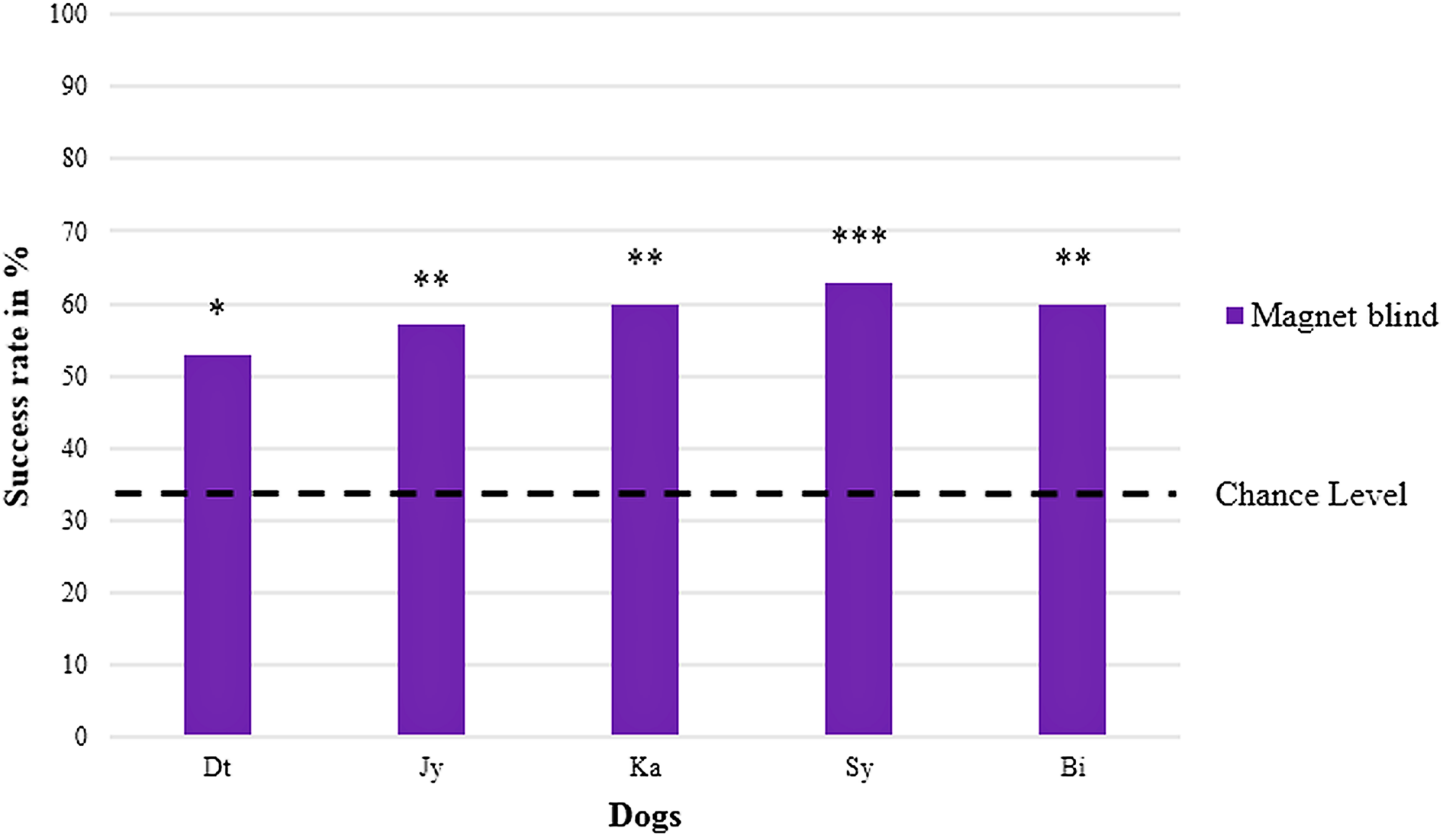
Percentage of correct choices in the blind replication experiment (magnet). Names are abbreviated by two letters; four males are on the left, one female on the right; both sexes are sorted by their abbreviated names; with *p < 0.05, **p < 0.01, and ***p < 0.001 (chance level at 33.3 %).

In the blind replication experiment, one of the retested dogs (Gy) refused to cooperate, meaning that it remained at the starting point and did not choose any of the jars when receiving the search command without a clear helping gesture of the experimenter. The five remaining dogs again showed a significant preference for the jar with the magnet (Chi-square test performed individually for each dog with all five *p*-values ≤ 0.02) with an average success rate of 59 ± 3% ranging between 53% and 63% (cf. Table 1, chance level at 33.3% and Fig. 3).

Here, the mean success rate of the five retested dogs differed significantly from the expected success rate at chance level (one sample *t*-test, *T* = 14.925, *p* < 0.001), too.

There were no significant differences between the dogs’ performances in test series A and its blind replication experiment (paired sample *t*-test, *T* = 0.497, *p* = 0.646, *n* = 5).

### Test series B

In test series B (“Find the food treat”), no dog chose the correct jar containing the food treat significantly above chance level. All 16 dogs made their choices randomly (Chi-square test performed individually for each dog with all 16 *p*-values ≥ 0.245) with an average success rate of 33 ± 5% ranging between 23% and 40% (cf. Table 1, chance level at 33.3%).

In this test series, the mean success rate of all 16 dogs did not differ significantly from the expected success rate at chance level (one sample *t*-test, *T* = −0.290, *p* = 0.776).

### Both tests in comparison

The dogs made significantly more often correct choices in test series A compared to test series B (paired sample *t*-test, *T* = 8.799, *p* < 0.001, *n* = 14), showing that they performed differently in finding the two objects (cf. Fig. 2).

The dogs did not show any observable sniffing behavior when making their decision, neither in test series A nor in test series B. They immediately started to move to the three jars after they had received the search command and made their decision after walking past the jar(s) very closely.

### Additional experiment with open jars without caps

All four dogs succeeded in finding the food treat under this condition (Chi-square test performed individually for each dog with all *p*-values < 0.001) with an average success rate of 84 ± 6% ranging between 77% and 90% (chance level at 33.3%). Moreover, in contrast to their behavior in the experiments with closed jars, in these experiments dogs displayed observable sniffing behavior before making their decision.

When comparing the dogs’ success rates in finding the food treat in the two conditions (closed jars and open jars) we found significant differences (paired sample *t*-test, *T* = 10.140, *p* < 0.01, *n* = 4).

## Discussion

Being aware that dogs might read their owner’s facial expressions or unintended signals to anticipate the desired behavioral response (McKinley & Sambrook, 2000; Range & Virányi, 2011; Virányi et al., 2004), we took care that the dogs had no eye contact either to their owners or the experimenters as soon as the decision-making process took place. The experimenter, who had to reward for correct choices within 0.5 s (Pryor & Chase, 2014), as well as the owner, were standing behind the dog when it decided on one of the jars (cf. Fig. 1).

The dogs were able to find the magnet but not the food treat, indicating that olfaction played only a marginal role (if any at all) in these tests. Only when the caps were removed from the jars the dogs were able to find the food treat and they displayed observable sniffing behavior before making their decision on one of the jars in these tests. On the basis of these findings, we suggest that the animals relied on different sensory modalities (olfaction or magnetoreception) depending on the current test condition (open jars or closed jars). It is unlikely that an experimenter bias has influenced the results, because we replicated the findings in a double-blind experiment, where not only the owner (and the dog) was blinded, but also the performing experimenter. Since the MF strength of the bar magnet decreased rapidly with distance, it was only at short distances (< 40 cm) that the field strength was in the range known to be relevant for animal orientation (> 1 µT). At the starting point (4 m distance) the created MF was below the detection limit of the magnetometer and also below the detection threshold reported for any animal yet. Directional changes of the dogs could be observed, at the closest, at a distance of approximately 2 m or nearer, which might mean that dogs are sensitive to weak MFs in the nT range as suggested by the previous alignment study (Hart et al., 2013b).

Generally, studies of magnetoreception using the paradigm of operant conditioning on magnetic cues are challenging (Freire & Birch, 2010; Madden & Phillips, 1987; Wiltschko & Wiltschko, 2007) because the animals have difficulties establishing a cognitive link between the task and the magnetic cue, which is presumably relevant in a different context (e.g., spatial orientation). For this reason, and because we performed no problem-solving tasks whose solution led to a direct reward, we had to monitor the dogs’ mood and fatigue constantly. Nevertheless, by conditioning animals, our study convincingly and for the first time, demonstrates that domestic dogs can detect a magnet. We note that the small sample size of *n* = 5 in the blind replication experiment constitutes a limitation. To extend the knowledge about magnetoreception in domestic dogs, for example, about the underlying mechanism or the perceptual threshold of magnetic anomalies, further experiments should involve controlled MFs in the Earth strength range using double-wrapped coils.

The question of the biological function(s) of magnetoreception in dogs has yet to be clarified. Apart from (anecdotal) reports about displaced dogs that found their way back home across long distances from unknown localities, there are also a few scientific records about their outstanding homing abilities, which might be supported by a magnetic sense. In World War 1 hundreds of dogs were trained to act as “British messenger dogs,” at which they had to find the way back to their owners through unknown terrains and across distances of several kilometers (Richardson, 1921). Subsequently, the first systematic studies on homing in dogs (and closely related canine species) were performed (Danner & Fisher, 1977; Harry, 1922; Henshaw & Stephenson, 1974; Phillips & Mech, 1970), refusing the "traditional" senses to be the sufficient explanation for these remarkable orientation skills. Being aligned with the MF in a certain way might help animals to store the coordinates as landmarks in their memory, calibrate their magnetic compass and/or compare their actual position with their cognitive map (Hart et al., 2013b; Phillips, Muheim & Jorge, 2010). This ability would be helpful not only for long-distance migration but also for spatial orientation at smaller scales (Begall, Burda & Malkemper, 2014; Hart et al., 2013a; Phillips, Muheim & Jorge, 2010). Notably, wolves, dogs’ ancestors, have enormously large home ranges (Kusak, Skrbinsek & Huber, 2005; Mattisson et al., 2013).

## Conclusions

In conclusion, we demonstrate that most of the tested dogs were able to find a hidden magnet significantly more often than expected by chance, whereas none of them was able to find a nonmagnetic food treat sealed in the closed jar beyond the probability given by chance. Based on these findings, we suggest that dogs find the magnet by detecting its MF. Considering the experimental design and the fact that the dogs were trained to respond directly and solely to the magnetic stimulus rather than a magnetic component of a multisensory task (e.g., “Find the way back home” or, in rodents, “Build a nest”), we have come to the conclusion that the animals perceived the magnet. These results indicate that the domestic dog can be added to the list of species which have already been demonstrated to show a spontaneous or learned response to artificial MFs that can be considered as magnetic anomalies (Denzau et al., 2011; Freire et al., 2012; Malewski et al., 2018; Thalau et al., 2007). The sensory basis of this perception and the role of MFs in dog behavior are unknown, but our findings may hopefully stimulate further studies of magnetoreception in mammals.

## Supplemental Information

10.7717/peerj.6117/supp-1 Supplemental Information 1 Raw data of test series A (magnet).

10.7717/peerj.6117/supp-2 Supplemental Information 2 Raw data of test series B (food treat).

10.7717/peerj.6117/supp-3 Supplemental Information 3 Raw data of blind replication experiment (magnet).

10.7717/peerj.6117/supp-4 Supplemental Information 4 Raw data of additional experiment (open jars).

10.7717/peerj.6117/supp-5 Supplemental Information 5 Top view of the blind replication experiment (preparation).

10.7717/peerj.6117/supp-6 Supplemental Information 6 Top view of the blind replication experiment (performance).

## Additional Information and Declarations

### Data Availability

The following information was supplied regarding data availability: Raw data are provided in the Supplemental Materials.
