# Supplementary figures and images for: Dogs can be trained to find a bar magnet
Source: PeerJ. 2018 Dec 17;6:e6117. doi: 10.7717/peerj.6117 (PMC6301327; doi:10.7717/peerj.6117)

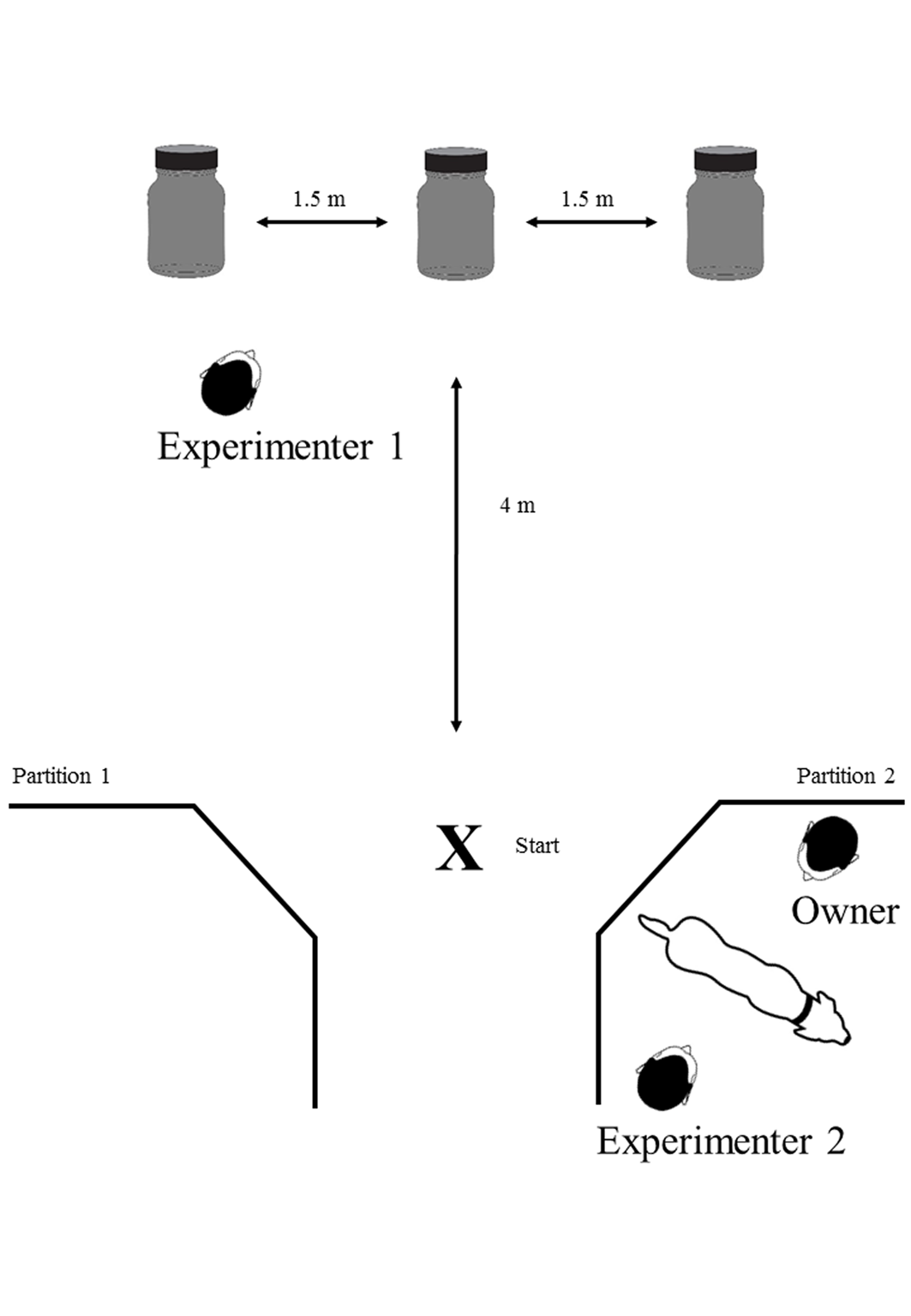

Supplement: Supplemental Information 5 [file peerj-06-6117-s005.png]

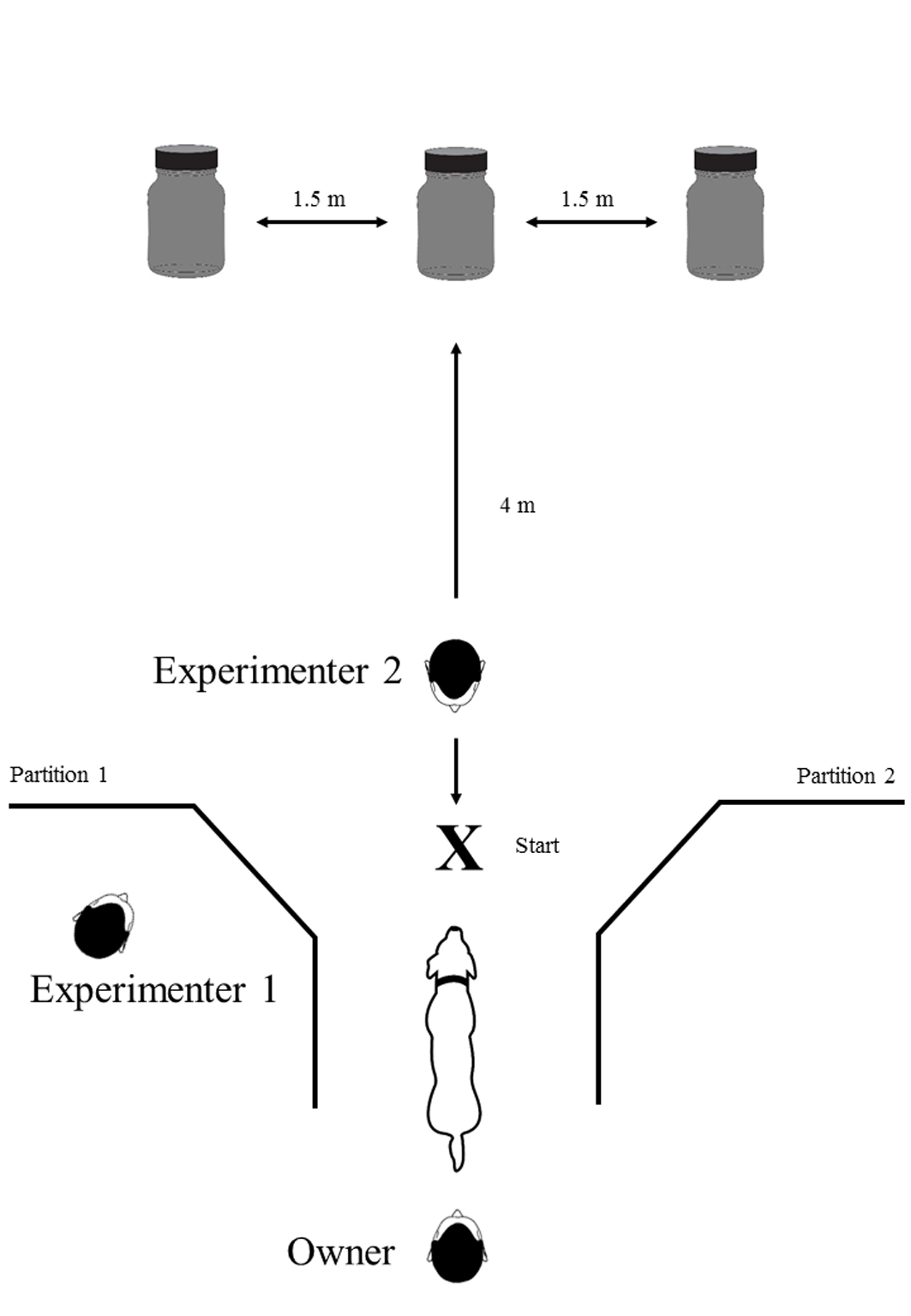

Supplement: Supplemental Information 6 [file peerj-06-6117-s006.png]
